# Improvements in access to malaria treatment in Tanzania after switch to artemisinin combination therapy and the introduction of accredited drug dispensing outlets - a provider perspective

**DOI:** 10.1186/1475-2875-9-164

**Published:** 2010-06-15

**Authors:** Sandra Alba, Manuel W Hetzel, Catherine Goodman, Angel Dillip, Jafari Liana, Hassan Mshinda, Christian Lengeler

**Affiliations:** 1 Dept. Epidemiology and Public Health, Swiss Tropical and Public Health Institute, Basel, Switzerland; 2 University of Basel, Basel, Switzerland; 3 Ifakara Health Institute, Ifakara, Tanzania; 4 Papua New Guinea Institute of Medical Research, Goroka, Papua New Guinea; 5 Department of Global Health and Development, London School of Hygiene and Tropical Medicine, London, UK; and Kenya Medical Research Institute-Welcome Trust Research Programme, Nairobi, Kenya; 6 Management Sciences for Health, Dar es Salaam, Tanzania; 7 Tanzanian Commission for Science and Technology, Dar es Salaam, Tanzania

## Abstract

**Background:**

To improve access to treatment in the private retail sector a new class of outlets known as accredited drug dispensing outlets (ADDO) was created in Tanzania. Tanzania changed its first-line treatment for malaria from sulphadoxine-pyrimethamine (SP) to artemether-lumefantrine (ALu) in 2007. Subsidized ALu was made available in both health facilities and ADDOs. The effect of these interventions on access to malaria treatment was studied in rural Tanzania.

**Methods:**

The study was carried out in the villages of Kilombero and Ulanga Demographic Surveillance System (DSS) and in Ifakara town. Data collection consisted of: 1) yearly censuses of shops selling drugs; 2) collection of monthly data on availability of anti-malarials in public health facilities; and 3) retail audits to measure anti-malarial sales volumes in all public, mission and private outlets. The data were complemented with DSS population data.

**Results:**

Between 2004 and 2008 access to malaria treatment greatly improved and the number of anti-malarial treatment doses dispensed increased by 78%. Particular improvements were observed in the availability (from 0.24 shops per 1,000 people in 2004 to 0.39 in 2008) and accessibility (from 71% of households within 5 km of a shop in 2004 to 87% in 2008) of drug shops. Despite no improvements in affordability this resulted in an increase of the market share from 49% of anti-malarial sales 2005 to 59% in 2008. The change of treatment policy from SP to ALu led to severe stock-outs of SP in health facilities in the months leading up to the introduction of ALu (only 40% months in stock), but these were compensated by the wide availability of SP in shops. After the introduction of ALu stock levels of the drug were relatively high in public health facilities (over 80% months in stock), but the drug could only be found in 30% of drug shops and in no general shops. This resulted in a low overall utilization of the drug (19% of all anti-malarial sales)

**Conclusions:**

The public health and private retail sector are important complementary sources of treatment in rural Tanzania. Ensuring the availability of ALu in the private retail sector is important for its successful uptake.

## Background

It is increasingly recognized that the Roll Back Malaria (RBM) Partnership target of '80% of malaria patients receiving effective treatment within 24 hours' cannot be achieved in sub-Saharan Africa unless anti-malarial drugs are made available outside the formal health sector [1-3]. The public health sector plays a central role in the delivery of key curative and preventative interventions for malaria in most African countries. However, health facilities alone do not guarantee satisfactory levels of access in countries where malaria is endemic. Surveys conducted between 2007 and 2008 in 11 African countries found that despite large increases in the number of anti-malarial treatments supplied through the public health sector, only 15% of children with fever were treated with artemisinin combination therapy (ACT) [4].

As a result, it has been argued that the delivery of anti-malarials needs to be supplemented by additional distribution mechanisms. This can be achieved through: 1) community case management and 2) strengthening the role of the private retail sector. The World Health Organization (WHO) is increasingly promoting the provision of anti-malarial drugs through community health workers with its Home Management of Malaria (HMM) strategy [5]. However, the WHO also recognizes the importance of private retailers [6] and various interventions aimed at improving their services have been developed and piloted in different country settings [7].

Reviews of the available literature reveal that shops are often preferred to health facilities as a first treatment action [8,9]. Retailers tend to be more accessible, have longer and more flexible opening hours, are willing to negotiate charges and offer credit, are more polite and friendlier and are generally perceived as being cheaper. Patients also seek treatment in the private sector out of necessity since public health facilities experience frequent stock-outs. However, the private retail sector is often poorly regulated in low and middle income countries. Common opportunistic practices include illegal stocking of prescription-only medicines, the use of unqualified staff and referral by health facility staff to private outlets in which they have a financial stake [10-12]. The advent of ACT compounds the problem further. In absence of regulation, people are likely to buy cheaper and less effective drugs from the private retail sector. Furthermore the misuse of ACT and the use of artemisinin monotherapies could contribute to the emergence and spread of artemisinin resistance.

In the past five years, two major interventions which directly affect access to malaria treatment have been implemented in Tanzania. Firstly, in 2002 the Tanzanian Food and Drug Administration (TFDA) registered a new class of drug shops known as accredited drug dispensing outlets (ADDOs). With over 8,000 outlets nation wide, drug shops are an important source of anti-malarial treatment in Tanzania, but unqualified staff invariably sells drugs they are not legally authorized to stock [13-15]. The aim of ADDOs is to address this poor state of regulation by upgrading existing drug shops (known as Part II) to properly operated outlets through a combination of dispenser training, financial incentives, accreditation and regulation [16]. Motivated by the experience of pilots conducted in the Ruvuma Region with support from the Bill and Melinda Gates Foundation (BMGF), the Ministry of Health and TFDA have planned the conversion of all Part II drug shops in the country by 2010 [17]

Secondly, in 2006 SP was abandoned in favour of artemether-lumefantrine (ALu, trade name Coartem^®^, Novartis AG) as first-line treatment for malaria due to high levels of resistance to SP. The actual introduction of ALu in health facilities was delayed until January 2007. ALu is currently unaffordable for most of the population because of the high market price of USD 8-10 for an adult dose. Thanks to support from the Global Fund to Fight AIDS, TB and Malaria (GFATM) the drug is provided free to all government health care facilities, where it should be made available free to children under the age of five and pregnant women; and at subsidized price of TSH 300 (USD 0.25) to other patients. Subsidized ALu was also made available in all ADDOs in the country by the end of 2007. Initial plans to upscale this scheme are likely to be superseded in 2010 by the Affordable Medicines Facility for malaria (AMFm). The AMFm aims to make ACT available at the cost of the previous generation of anti-malarials by negotiating with the manufacturers to reduce the price of their ACT and subsidizing them heavily for purchase by specified national importers. It is expected that drugs will be available at about USD 0.20-0.50 to patients. Tanzania and Zanzibar are among the 10 countries invited to pilot a first phase of this subsidy as from mid 2010 [18].

This study presents a unique set of longitudinal data on availability, accessibility, costs and uptake of anti-malarial treatment in both the private retail and public health sectors. The data were collected between 2004 and 2008 in two districts of the Morogoro Region in the frame of the ACCESS programme, which aims at improving and understanding access to malaria treatment [19]. The results presented here are complemented by treatment-seeking surveys which evaluated changes in treatment outcomes (i.e. the user perspective) [20]. This experience is of great relevance to other settings, as ACT is being adopted throughout most of Africa as first-line treatment for malaria and ACT subsidies will be introduced widely in the private sector through the AMFm.

## Methods

### Study setting

**Figure 1:**
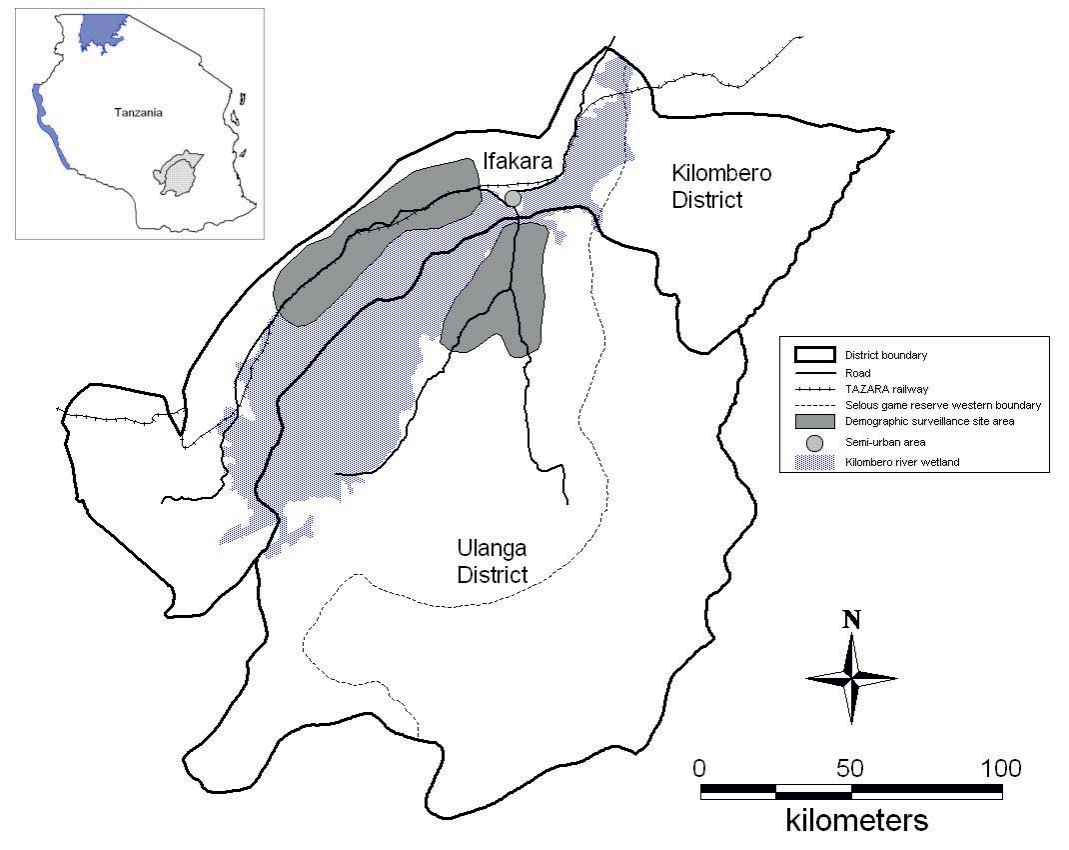
**Map of Kilombero and Ulanga Districts showing Ifakara Town and the Demographic Surveillance System (DSS)**. Source: [24].

The study was carried out between 2004 and 2008 in the rural villages of the Kilombero and Ulanga Demographic and Surveillance System (DSS) and in the semi-urban setting of Ifakara town in south-central Tanzania (Figure 1). The rural DSS area covers 25 villages (13 in the Kilombero District and 12 in Ulanga District). The population in mid-2004 was 73,977 and increased at an average rate of 5% per year, resulting in a population of just over 92 203 in mid-2008. The population of Ifakara town was 45,518 in 2002 according to the last national census [21].

Malaria transmission in the area is intense and perennial with differences between the rural and semi-urban settings. Overall transmission has been declining over the past 10 years. A study conducted in the area between 2001 and 2003 reported an entomological inoculation rate (EIR) of 349 infective bites per person per year (ib/p/y) [22], but according to recent data it has declined to 81 ib/p/y (Russell *et al*., unpublished data). EIR data for Ifakara town suggest that the transmission rate is about a log order smaller than in the surrounding rural areas [23]. Treatment-seeking surveys carried out in 2004, 2006 and 2008 within the frame of the ACCESS programme found on average twice as many fever cases in the rural areas compared to Ifakara town (Alba *et al.*, unpublished data).

There are six health facilities (three public dispensaries, one public health centre and two faith-based dispensaries) in the Kilombero rural DSS area and eight health facilities (five public dispensaries, one health centre and two faith-based dispensaries) in the Ulanga rural DSS area. The Designated District Hospital in Ifakara serves as a referral point for all the facilities in the study area. There is only one Part I pharmacy in the study area, which is located in Ifakara town. The area has been described in more detail elsewhere [19,24].

Data collection activities are summarized in Table 1 and described in detail below. The routine monitoring in health facilities and the shop censuses, combined with DSS data, provided information on availability and accessibility of sources of anti-malarial drugs. The retail audits provided data on prices and uptake of anti-malarial drugs.

**Table 1:** Summary of data collection activities

*Activity*	*Collection period* (*month.year*)	*Sample*	*Location*
**Routine monitoring in health facilities**			
1) Collection of monthly data on availability of anti-malarial drugs in all public health facilities from ledger books	1.2005 - 12.2008	10	Rural villages (DSS)
2) GPS location of each health facility			

**Shop census**			
Census of all shops potentially selling drugs providing quantitative data on	5.2004 - 7.2004	758	Rural villages (DSS) and Ifakara Town
	3.2005 - 7.2005	790	
1) Availability of shops selling anti-malarial drugs	4.2006 - 7.2006	878	
2) Availability of anti-malarial drugs	4.2007 - 8.2007	1074	
3) GPS location of each shop	4.2008 - 9.2008	1082	

**Retail audits**			
1) Measurement of anti-malarial sales during a two-week period in all outlets (shops and health facilities)	4.2005 - 7.2005	78	Rural villages (DSS)
2) Retail prices of each anti-malarial drug	4.2008 - 7.2008	66	

### Routine monitoring in health facilities

Every other month a team of trained field workers visited the ten public health facilities in the rural DSS area (four in Kilombero and six in Ulanga) to collect end-of-month anti-malarial drug stock data from the Health Management and Information System (HMIS) ledger books, using validated data collection tools. Data could not be collected from the faith based facilities as they are not legally required to record drug stock in ledger books, rendering systematic collection of the data difficult. In 2004 the GPS locations of all the health facilities in the area were recorded with a hand-held GPS unit (Garmin^® ^e-Trex, Garmin^® ^Ltd).

The percentage of facility-months in stock of a given anti-malarial over the year was calculated as an indicator of drug availability. First, an average estimate of yearly drug availability was calculated for each drug and each health facility. Unfortunately, the data on drug availability were missing in the ledger books for many months. Anecdotal evidence suggests that facility staff tended not to fill in the entry on end-of-month stocks when the drug was not available. As a result, and in the absence of any other information, months with missing data were considered as "out of stock". If a health facility had more than 25% missing values it was excluded from the average to avoid skewing estimates of yearly drug availability towards unrealistically low values. Then an average estimate of anti-malarial availability for the area was calculated by averaging the yearly estimates from all the health facilities. The estimate was deemed inconclusive if more than 25% facilities had to be excluded from the average estimate.

The health facility data were complemented with population numbers and household locations to provide standardized indicators of availability and accessibility of health facilities. Population counts down to village level were available for every year between 2004 and 2008 from the DSS database. GPS positions were available from the DSS database for 85% (16141/18840) of households in 2004 and 90% (18580/20579) of households in 2008. An indicator of availability of health facilities was constructed as the number of shops per 1,000 population, and an indicator of accessibility as the average distance between households and the nearest health facility.

### Shop census

Repeated cross sectional shop censuses were carried out every year in the rural DSS area and in Ifakara town. All shops potentially selling drugs (general stores, kiosks, Part II drug stores and ADDOs) were visited by a team of nine trained field workers who recorded the outlet's stock and location using tools and methodology developed and previously applied in the area [3,25]. The only Part I pharmacy was not visited. The primary aim of the census was to assess the type and brand of drugs available in each type of outlet. The analysis of the baseline study in 2004 was published by Hetzel *et al*. [25] and the results presented here provide a longitudinal assessment of the changes between 2004 and 2008. The secondary aim of the census was to assess shopkeepers' knowledge about signs, symptoms and treatment of malaria. Results will be presented elsewhere (Dillip *et al*., in preparation).

Data were collected using a structured questionnaire and the location of each shop was recorded with a hand-held GPS unit. The field work was overseen by the same supervisor in all years. The supervisor visited all the village and hamlet leaders prior to the censuses to inform them and to answer any queries. Every year, field interviewers were provided with an inventory of all the outlets identified in the previous year's census and were instructed to visit every outlet on the list, as well as any new outlet they encountered. Random quality checks were performed by a research scientist who visited the field sites shortly after the field workers.

Estimates of availability and accessibility of shops stocking drugs were constructed using the methodology outlined for health facilities. Population counts were estimated for Ifakara town by adjusting the 2002 census count with the annual growth rate in the rural DSS area.

### Retail audits

Two retail audits of anti-malarial drugs were conducted in the rural DSS during the shop censuses of 2005 and 2008. The methodology developed by Goodman *et al *[26,27] was applied. The retail audit included all outlets selling or dispensing drugs, i.e. all health facilities (public and faith-based) and drug shops (Part II drug shops and ADDOs), as well as the few general shops found to stock anti-malarial drugs during the shop census. The aim of the study was to estimate total anti-malarial sales volume in the study area and to compare the share of sales of each anti-malarial drug across each type of outlet.

The retail audit data were collected over two consecutive visits two weeks apart. On the first visit, which coincided with the shop census visit, field interviewers recorded stock levels for each anti-malarial drug. On the second visit they recorded stock levels and any deliveries since their last visit, as well as any drugs that had been given out for any other reason e.g. thrown away or returned to wholesalers. Two weeks was considered a reasonable recall period for wholesale deliveries. The exact number of tablets, bottles or vials was counted where possible. When tablets were kept in tins the following information was collected in order to estimate the number of tablets present: the height of the tablets in the tin when full, the height of tablets on the day of visit and the number of tablets in a full tin. In addition the reported retail price per single unit (be it a tablet, bottle or vial) was recorded. The retail audit was carried out once a year (in approximately the same months) as no significant seasonal differences were found in the previous study in which two surveys per year were conducted [27].

Daily sales were calculated or estimated for each anti-malarial. Sales between the two visits were calculated as: Sales = (total at first visit) + (deliveries between first and second visit) - (stocks thrown away or transferred to other shops/facilities) - (total at second visit). The number of tablets, bottles of syrup and vials was converted into the equivalent number of adult doses in order to calculate the total anti-malarial sales of different types of anti-malarials. Daily sales were calculated by scaling down up or down pro rata according to the number of days between the two audit visits. If total sales volumes could not be calculated (because drug levels were not recorded on the first or the second visit), they were estimated by a multiple imputation. With this statistical technique missing values are imputed several times based on random draws from the conditional distribution of the missing observations given the observed data and covariates (drug levels on the first or second visit, type of outlet, type of drug and drug formulation). In this case three draws were considered enough and the average between the three imputations kept for analysis. Multiple imputation was deemed appropriate as there were no indications that the data were not missing at random.

Reported drug prices were adjusted for inflation to reflect actual changes in purchasing power. The 2005 drug prices were inflated to real 2008 values on the basis of consumer price indices [28]. An inflation factor of 1.25 was derived from the mean yearly inflation values published by the Tanzania National Bureau of Statistics (NBS) [29].

### Data entry and analysis

Data were double entered using Microsoft FoxPro and Microsoft Access (Microsoft Corp. Seattle, USA) and checked for coding errors and consistency. Intercooled Stata 10 (Stata Corp., College Station, TX, USA) was used for data management and analysis. Logistic and Poisson regressions provided odds ratios (OR) and incidence rate ratios (IRR) to estimate changes over time or differences between areas. ORs and IRRs indicate an average increase per year between the years of observation ("year" is entered in the regression as a linear variable) unless it is explicitly stated that the increase is not linear and is from a given year to another ("year" is entered as a categorical variable). Differences in medians between years were tested using a two-sample Wilcoxon rank-sum (Mann-Whitney) test. Distances were calculated with ArcMap Version 9.1 (ESRI Inc.)

### Ethics

The National Institute for Medical Research of the United Republic of Tanzania (NIMR/HQ/R.8a/Vol.IX/236, 16^th ^September 2003) granted ethical clearance for the study Shop keepers and health facility staff gave informed consent to participating in the study and were given feedback of the study results.

## Results

### Availability and accessibility of anti-malarials in public health facilities

**Figure 2:**
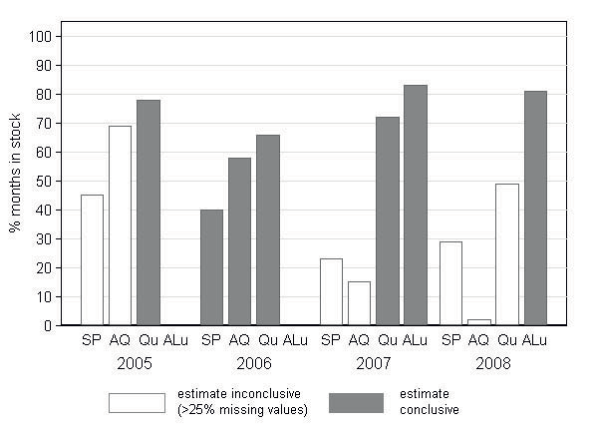
**Availability of anti-malarial drugs in public health facilities in the rural DSS area**. Note ALu was introduced in January 2007.

Drug availability data were collected for ten facilities over four years for four drugs (two years for ALu) i.e. 1,680 facility-months but only 1,380 facility-months of observation could be included in the analysis. As a result out of the 14 yearly overall availability estimates were deemed inconclusive (Figure 2).

Towards the end of 2006, just before the introduction of to ALu, health facilities experienced severe stock-outs of SP. On average throughout the year the drug was only available 40% (38/96) of the months with three facilities reporting stockouts for more than 10 months. This was mainly due a delay in the introduction of ALu without additional SP orders to compensate. The availability of quinine and amodiaquine was much higher as the drugs were reported to be available 66% (78/120) and 58% (70/120) of the time, respectively (Figure 2). However, four facilities were out of stock of all three anti-malarials for a mean of 2.8 (sd = 1) months of the year. Since the introduction of ALu availability of anti-malarial drugs has greatly improved. In 2007 and 2008 ALu was available for more than 80% of the months of the year (90/108 in 2007 and 88/108 in 2008).

The availability and accessibility of health facilities was only slightly affected by population growth. The availability of public health facilities decreased slightly but not significantly. In 2004 there were 0.13 facilities per 1,000 people (10/73,977) and in 2008 this figure declined to 0.11 (10/92,203) (IRR = 0.81 p = 0.712 from 2004 to 2008). The median distance from a household to a facility marginally increased from 2.25 km (IQR = 4.31) to 2.37 km (IQR = 4.75) (z = -5.388 p < 0.001) and the proportion of households located within 5 km of a health facility decreased from 73% (11,858/16,141) to 70% (12,990/18,580) in 2008 (OR = 0.90 p < 0.001 from 2004 to 2008).

### Availability and accessibility of malaria treatment in drug shops

The number of shops included in the census increased on a yearly basis (Table 2). Every year general shops (permanent structures and kiosks) consistently accounted for 95% of all shops potentially selling drugs, while the rest were Part II drug shops and ADDOs. The number of Part II drug shops decreased considerably after 2006 as they were progressively upgraded to ADDOs. Overall the number of drug shops (Part II drugs stores and ADDOs) increased from 34 to 59 between 2004 and 2008.

**Table 2:** Number of shops censused, by type

		*2004*	*2005*	*2006*	*2007*	*2008*
**General shops (including kiosks)**	Kilombero DSS	343	293	313	385	442
	Ulanga DSS	201	199	180	213	201
	Ifakara town	185	256	341	421	380
	**Total**	**729**	**748**	**834**	**1019**	**1023**

**Part II drug stores**	Kilombero DSS	16	22	17	8	6
	Ulanga DSS	3	9	4	2	3
	Ifakara town	10	11	17	1	1
	**Total**	**29**	**42**	**40**	**11**	**10**

**ADDOs**	Kilombero DSS	0	0	0	18	22
	Ulanga DSS	0	0	4	8	7
	Ifakara town	0	0	0	17	20
	**Total**	**0**	**0**	**4**	**43**	**49**

**Total**	Kilombero DSS	359	315	330	412	470
	Ulanga DSS	204	208	190	223	211
	Ifakara town	195	267	358	439	400
	**Total**	**758**	**790**	**878**	**1074**	**1083**

**Figure 3:**
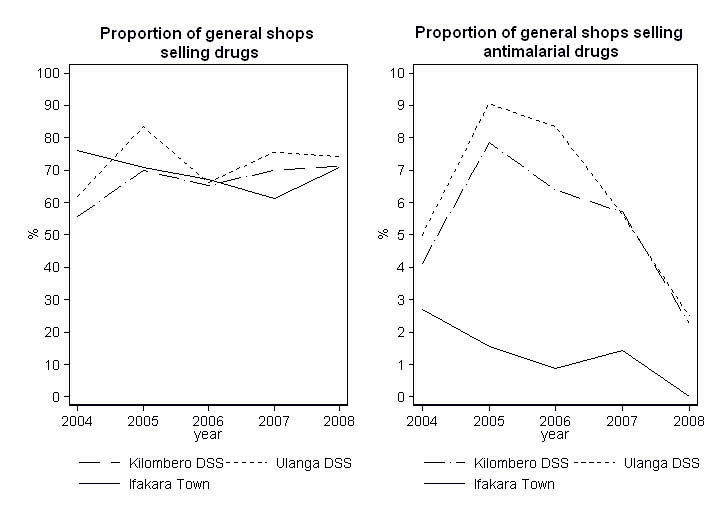
**Proportion of general stores selling any drugs and anti-malarials.** Note: vertical axis differs between two graphs.

Across all years a high proportion of general shops stocked some type of drug and a high proportion of drug shops stocked anti-malarials, but a varying and much smaller proportion of general shops stocked anti-malarials (Figure 3). Between 2004 and 2008 an average of 68% of general shops stocked drugs (mainly antipyretics), with no significant differences between districts or between the rural areas and Ifakara town. However, few of these were found to sell anti-malarial drugs. The proportion of general shops selling anti-malarials never exceeded a peak of 9% (18/199 in Ulanga and 23/293 in Kilombero) in 2005 in the rural DSS villages and 3% (5/185) in Ifakara in 2004 and decreased considerably thereafter to 3% (5/501 in Ulanga and 10/442 in Kilombero) in the rural DSS and to 0% (0/380) in Ifakara town in 2008 (DSS IRR = 0.87 p = 0.018, Ifakara IRR = 0.64 p = 0.013 between 2004 and 2008). The proportion of drug shops (Part II and ADDOs) storing anti-malarial drugs was 86% (31/36) in 2004, 100% (42/42) in 2005, 84% (37/44) in 2006 and over 98% in 2007 (54/55) and 2008 (58/59).

**Figure 4:**
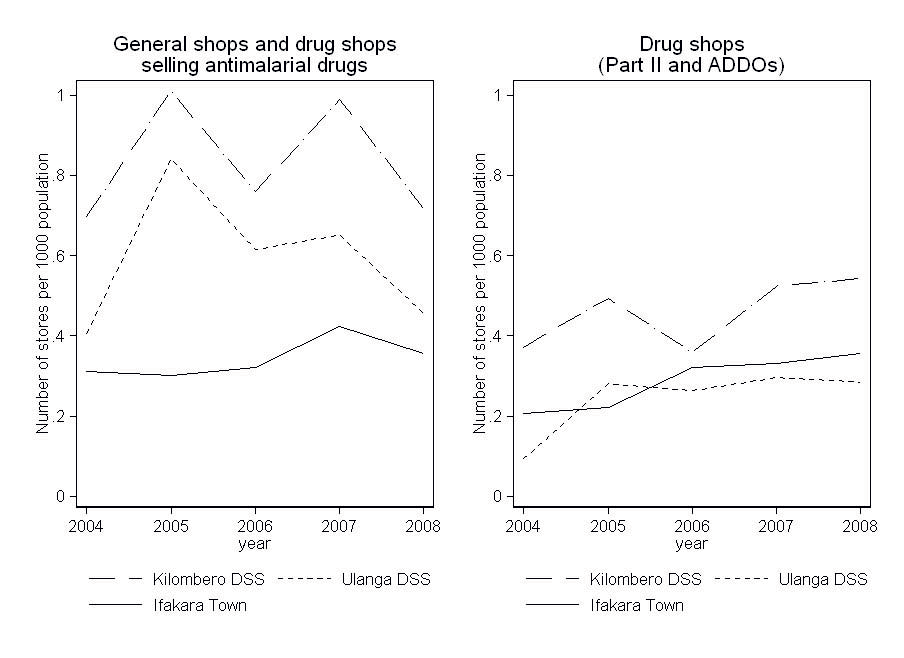
**Availability of retail outlets selling drugs, per 1000 population**.

There is evidence of greater availability of drug shops, even accounting for population growth. While the absolute number of anti-malarial retail points (drug shops and general shops) increased over the study period from 58 in 2004 to 74 in 2008, the number of shops per 1,000 people did not change significantly over the years and fluctuated around 0.73 shops per 1,000 people in rural DSS areas and 0.34 in Ifakara town (Figure 4). In contrast, the availability of drug shops alone did increase over the years, although not linearly, from 0.23 (29/123260) shops per 1,000 people in 2004 to 0.39 (59/151,260) in 2008 (IRR = 1.72 from 2004 to 2008 p = 0.019). Average rates mask differences at district and village level. The Kilombero rural DSS generally had higher availability of both general shops selling drugs (IRR = 0.71 p = 0.005) and drug shops (IRR = 0.53, p = 0.001) compared to the Ulanga rural DSS. Moreover, half of the 14/25 villages which did not have drug shops in 2004 still did not have any in 2008. Three of these did not have any health facilities either, and this accounts for 6% (5442/92203) of the total rural DSS population.

**Figure 5:**
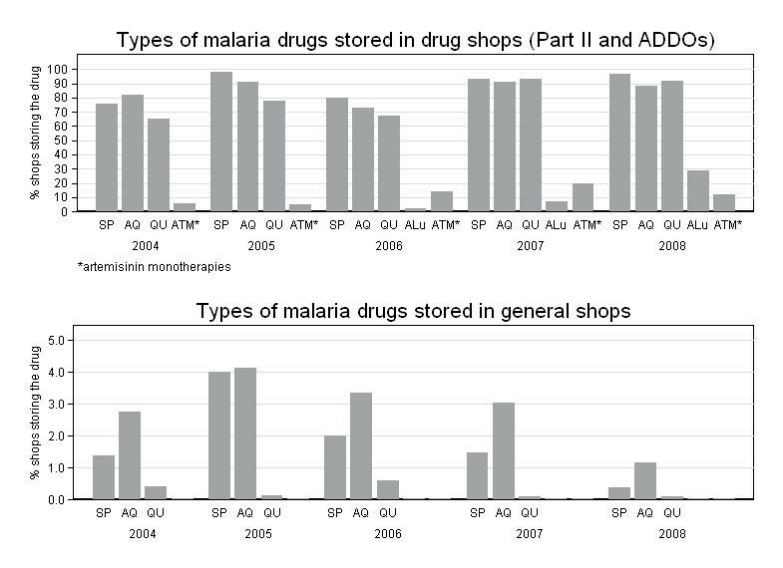
**Availability of different anti-malarial drugs in private retail outlets.** Note: vertical axis differs between two graphs.

Amodiaquine and SP were the most commonly available anti-malarials in both general shops and drug shops (Figure 5). In drug shops, the anti-malarial most commonly available was SP (90% of shops i.e. 210/234 over the study period), closely followed by amodiaquine (86% i.e. 200/234) and quinine (80% i.e. 187/234). ALu was available in 2% (1/43) of the drugs stores in 2006 (unsubsidized), 7% (4/54) in 2007 and 29% (17/58) in 2008. Interestingly, chloroquine could still be found in 2004 and in 2005 in one drug shop in a remote village of the Kilombero rural DSS area. Figure 5 gives a break down by year of these figures.

While artemisinin monotherapies were mainly found in the drug shops of Ifakara, ALu was also available in the rural areas. The proportion of shops storing artemisinin monotherapies grew until 2007, when up to 11 out of 55 (20%) shops stored drugs containing either artesunate, artemether or dihydroartemisinin (Figure 5). In 2008, this proportion decreased to 12% (7/59), probably as a result of tighter national and international regulations on the distribution of artemisinin monotherapies. However, it is worth noting that these artemisinin derivatives could be found in Ifakara but hardly reached the more rural villages, most likely because of their very high prices. ALu tended to be more available in Ifakara than in rural areas (Ifakara 8/13 i.e. 38.1% of drug shops stored ALu, Kilombero DSS 7/27 i.e. 25.9%, Ulanga DSS 2/10 i.e. 20%).

**Figure 6:**
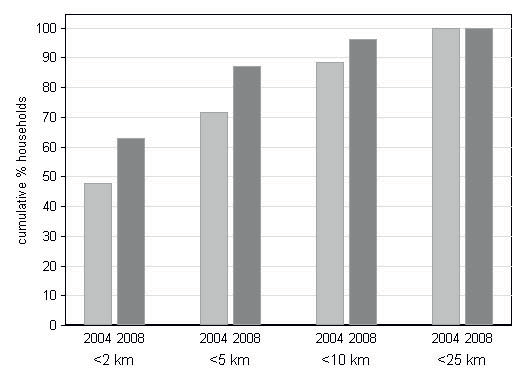
**Cumulative percentage of households within a given distance to the nearest drug shop**.

Drug shops became more accessible after the introduction of ADDOs. Across all years drug shops were more likely to be found in the most populous areas, but different patterns were noted for ADDOs and drug shops. Both in 2004 and in 2008 a significant association was found between the presence of any drug shop in a village and the size of the village (2004 OR = 2.6 for every 1,000 increase in population p = 0.016, 2008 OR = 2.6 p = 0.048). However, in 2008 the association between the presence of an ADDO in a village and the size of the village was much smaller (OR = 1.7 p = 0.077) suggesting that ADDOs were also opened purposefully in less populous areas. This resulted in an increase in the number of households located within 5 km from a drug shop from 71.5% (3,848/16,141) to 87% (11,662/18,580) (OR = 5.38 p < 0.0001 from 2004 to 2008) (Figure 6). The median distance from a drug shop to a household nearly halved during the study period, decreasing from 2.2 km (IQR = 4.8) to 1.2 km (2.6) (z = 32.234 p < 0.0001).

### Sales of anti-malarial drugs in shops and health facilities

In 2005 a total of 366 anti-malarial products were followed from 77 retail outlets (31 drug stores, 37 general stores, five kiosks and 14 health facilities). In 2008 a total of 409 products were followed from 66 outlets (10 drug stores, 12 general stores, three kiosks, 27 ADDOs and 14 health facilities). Sales levels could not be calculated and were estimated for 6% (44/775) items because drug levels were not recorded either in the first or in the second visit (see methods).

**Figure 7:**
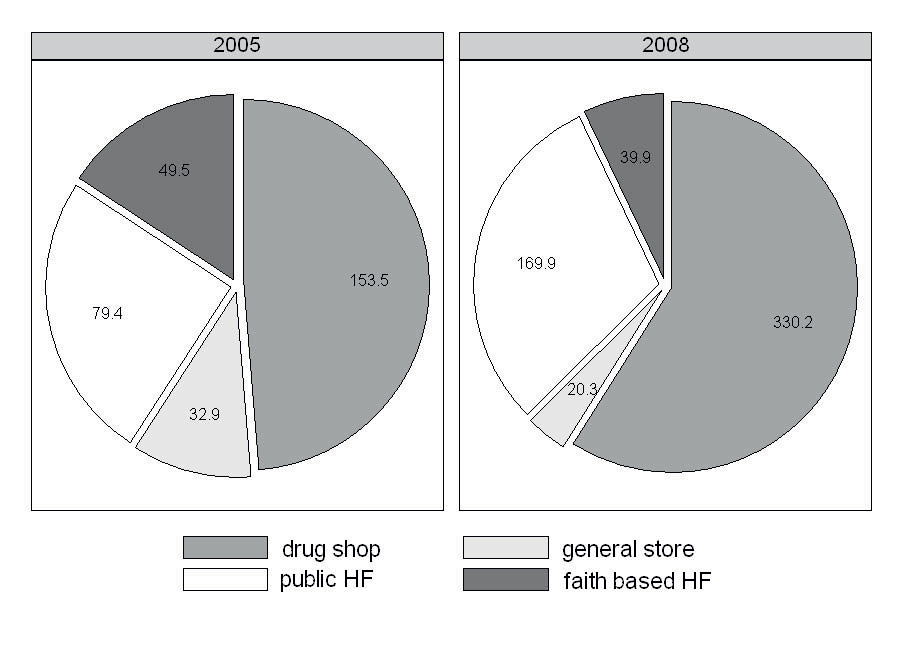
**Share of total anti-malarial sales through each outlet type**.

The analysis of daily sales revealed a near doubling in the number of anti-malarial doses dispensed between 2005 and 2008 and the increasing importance of drug shops as a source of anti-malarials. An estimated 315 equivalent adult anti-malarial doses were dispensed per day from all facilities and shops in the rural DSS areas in 2005 while 560 doses were dispensed in 2008 (78% increase). The proportion of anti-malarial drugs sold in drug shops increased from 49% (153.5/315.3) in 2005 to 59% (330.2/560.3) in 2008 (OR = 1.51 p = 0.036) (Table 3 and Figure 7). The increase in the use of drug shops was not at the expense of public health facilities, but at the expense of general shops and faith based facilities. Indeed, the proportion of doses sold in general shops and faith based health facilities decreased but there was no significant difference in terms of anti-malarials dispensed in health facilities (79.4/315.3 i.e. 25% in 2005 vs. 169.9/560.3 i.e. 30% in 2008 OR = 1.29 p = 0.115)

**Table 3:** Number of equivalent adult doses sold per day in all shops and health facilities (HF) in the DSS rural areas and share out of total in the type of outlet

	*2005*					*2008*				
	*Public HF*	*Faith Based* *HF*	*Drugs shops*	*General shops*	*Total*	*Public HF*	*Faith Based HF*	*Drugs shops*	*General shops*	*Total*
Amodiaquine	22.4 (28.2%)	21.0 (42.4%)	43.4 (28.3%)	10.8 (32.8%)	97.6 (30.9%)	64.7 (38.1%)	9.6 (29.2%)	62.1 (18.8%)	4.3 (21.2%)	140.7 (25.1%)
Chloroquine			0.9 (0.6%)		0.9 (0.3%)					-
SP	54.9 (69.2%)	26.8 (54.2%)	97.9 (63.8%)	21.9 (66.6%)	201.5 (63.9%)	26.6 (15.7%)	7.9 (24.0%)	233.6 (70.7%)	15 (78.8%)	284.1 (50.7%)
Quinine	2.1 (2.6%)	1.7 (3.4%)	11.3 (7.3%)	0.2 (0.6%)	15.3 (4.9%)	7.5 (4.4%)	7.5 (22.8%)	16.1 (4.9%)	> 0.1 (> 0.1%)	31.1 (5.1%)
ALu	-	-	-	-	-	71.1 (41.8%)	14.8 (50%)	18.1 (5.5%)		104.0 (18.6%)
Artemisinin monotherapy	-	-	-	-	-	0.1 (> 0.1%)		0.3 (> 0.1%)		0.3 (> 0.1%)

**Total**	**79.4**	**49.5**	**153.5**	**32.9**	**315.3**	**169.9**	**32.9**	**330.2**	**20.3**	**560.3**

The sales of SP remained very high in 2008 despite the change of treatment policy to ALu. In 2005 64% of all anti-malarial sales consisted of SP and in 2008 it still accounted for 51% of all anti-malarial sales. ALu only accounted for 19% of total sales in 2008, 68% (71.1/104.0) of which were in public health facilities, 14% (14.8/104.0) from faith based health facilities and 17% (18.1/104.0) from drug shops.

Prices were generally higher in drug shops where they also increased considerably over time. Prices of anti-malarials were compared between 2005 and 2008 in drug shops and public health facilities only (Table 4) although six out of 10 health facilities did not provide any pricing information. In 2005 health facilities reported selling SP adult doses at an average of USD 0.25 (USD 0.32 in 2008 prices) whereas it was sold for USD 0.37 (USD 0.45 in 2008 prices) on average in drug shops. In 2008 two of the facilities which provided prices reported dispensing ALu packs for children under the age of eight free, whereas two reported selling them for USD 0.25. Adult ALu and SP doses were reported to be sold for USD 0.25 in health facilities. Both SP and ALu were much more expensive in shops where sellers reported selling an ALu dose for USD 1.27 and SP doses for USD 0.67. Adjusting for inflation these price differences translate into a 20% decrease in the price of the first-line treatment in public health facilities. In drug shops prices increased by 51% when comparing the price of SP in 2005 and in 2008 and by 84% when comparing the price of SP in 2005 and the price of ALu in 2008.

**Table 4:** Reported retail price in Tanzanian Shillings (TSH) in drug shops and public Health Facilities (HF) of a single adult anti-malarial dose - unless otherwise specified (USD 1 = TSH 1180 in 2008 and USD 1 = TSH 1130 in 2005)

	*2005*				*2008*			
	*Drugs shops*		*Public HF*		*Drug Shops*		*Public HF*	
	*N*	*Median * *(IQR)*	*N*	*Median * *(IQR)*	*N*	*Median * *(IQR)*	*N*	*Median * *(IQR)*
Amodiaquine								
Tablets	35	0.35 (0.35 to 0.94)	4	0.35 (0.32 to 0.44)	31	0.42 (0.42 to 0.67)		-
syrup ^1^	36	0.72 (0.62 to 0.83)	1	0.25 (0.25 to 0.25)	38	0.99 (0.78 to 0.99)		-

Chloroquine tablets	1	0.26 (0.26 to 0.26)		-		-		-

SP	100	0.37 (0.26 to 0.79)	5	0.26 (0.26 to 0.26)	103	0.67 (0.25 to 1.27)	4	0.25 (0.13 to 0.25)
Quinine								
Tablets	26	1.85 (1.85 to 1.85)	3	1.85 (1.85 to 1.85)	28	3.56 (3.56 to 3.56)	3	2.85 (1.78 to 3.56)
syrup ^1^	15	3.35 (2.79 to 4.18)		-	38	5.34 (5.34 to 5.34)		
Injection ^2^	18	9.29 (7.43 to 9.29)	5	7.43 (5.58 to 9.28)	30	8.90 (8.90 to 0.68)	2	8.89 (8.89 to 8.89)

Artemether-Lumefantrine								
3 m - 3 y		-		-	8	0.42 (0.42 to 1.06)	4	0.05 (0 to 0.18)
3 - 8 y		-		-	9	0.42 (0.42 to 0.84)	3	0.25 (0 to 0.25)
8 - 12 y		-		-	8	1.27 (1.14 to 6.99)	4	0.13 (0 to 0.13)
12 y +		-		-	7	1.27 (1.27 to 1.69)	4	0.25 (0.13 to 0.25)

Artemisinin monotherapies		-		-	2	4.68 (4.29 to 5.08)		

^1 ^dose for a child between 1 and 3 years of age

^2 ^calculated assuming a full course of quinine is taken intra-muscularly for 7days

## Discussion

The findings presented here provide an independent assessment of the changes in access to anti-malarial treatment in the private and the public sector after the roll-out of ADDOs and the introduction of ACT in the Kilombero and Ulanga Districts of Tanzania. A framework recently published by Obrist *et al*. views access as the degree of fit between providers' services and users' means along the five dimensions of availability, accessibility, affordability, adequacy and acceptability [30]. Appropriate levels of access lead to utilization of treatment and eventually to improved health, provided the treatment is of high quality. This study has concentrated on the provider side of access and changes in levels of availability, accessibility and affordability of treatment in both the private health sector and the commercial retail sector are summarized in Table 5 and Table 6. These access indicators can be viewed as outputs in the standard evaluation terminology [31], whereas the treatment-seeking surveys conducted alongside this study focused on treatment outcomes [20]).

**Table 5:** Summary of changes in access to malaria treatment from the public health sector

*Dimension*	*Indicator*	*Difference*	*Change*
Availability	Facilities per 1000 population	**0.13 **in 2004 vs. **0.11 **in 2008 (p = 0.712)	
	Proportion of months in stock of anti-malarial drugs	Data not conclusive	
	Proportion of months in stock of the first-line anti-malarial	**40% **SP stock in 2006 vs. **81% **ALu stock in 2008 (p < 0.001)	+

Affordability	Median price of an adult anti-malarial dose	SP TSH**375 **(in 2008 prices) in 2005 vs. SP TSH **300 **in 2008 and ALu TSH**300 **in 2008	-

Accessibility	Population within 5 km from a facility	**73% **in 2004 to **70% **in 2008 (p < 0.001)	-
	Median distance from a household to a facility	**2.2 km **in 2004 vs. **2.3 km **in 2008 (p = 0.457)	

Utilization	Number of equivalent adult doses dispensed per day	**79 **in 2005 vs. **170 **in 2008	+
	Share of total sales through public health facilities	**25% **in 2005 vs. **30% **in 2008 (p = 0.115)	

**Table 6:** Summary of changes in access to malaria treatment from the private retail sector

*Dimension*	*Indicator*	*Difference*	*Change*
Availability	Shops per 1000 population	**0.24 **in 2004 vs. **0.39 **in 2008 (p = 0.05)	+
	Proportion of licensed drug shops storing anti-malarials	**86% **in 2004 vs. **98% **in 2008 (p = 0.019) but not a constant trend over the years	
	Proportion of licensed drug shops storing the first-line anti-malarial	**86% **selling SP in 2004 vs. **29% **selling ALu in 2008 (p < 0.001)	+

Affordability	Median price of adult anti-malarial dose	SP TSH**493 **(in 2008 prices) in 2005 vs. SP TSH**800 **in 2008 and ALu TSH**1500 **in 2008	-

Accessibility	Population within 5 km from a shop	**72% **in 2004 vs. **87% **in 2008 (p < 0.001)	+
	Median distance from a household to a shop	**2.2 km **in 2004 vs. **1.2 km **in 2008 (p < 0.001)	+

			

Utilization	Number of equivalent adult doses dispensed per day	**154 **in 2005 vs. **330 **in 2008	+
	Share of total sales through drug shops	**49% **in 2005 vs. **59% **in 2008 (p = 0.036)	+

Overall there has been a great improvement in access to malaria treatment. The number of anti-malarial doses dispensed nearly doubled over the three years of observation. Major gains were made in the private retail sector in terms of availability and accessibility of treatment, but availability of drugs also improved in health facilities following the introduction of ALu. The treatment-seeking surveys [20] conducted in the study area found that this was accompanied by an increase in the promptness of treatment. The data show that the proportion of anti-malarial doses taken within 24 hours of symptom onset increased from form 80% in 2004 to 93-97%.

There was a stark improvement in the availability of the first-line treatment in public health facilities over the study period. In 2008 health facilities were in stock of ALu in over 80% of the months of the year compared to 40% in stock of SP in 2006. It is worth noting that the value of 80% ALu stock was determined using a very conservative method of estimation whereby missing values were considered out of stock (see methods). A more liberal calculation which excludes missing values from any calculation yields values of 94% stock in 2007 and 91% in 2008. This presents a noteworthy improvement compared to previous years. There is still room for improvement though, and ACCESS is continuously working with the District Health Management Teams to close the gap. The severe stock-outs of SP in 2006 during the months leading up to the introduction of ALu showed the importance of having an alternative source of anti-malarials in the community.

The high levels of stock of ALu in 2007 and 2008 coincided with the implementation of a "push" system of delivery, which was implemented as a temporary measure for the distribution of ALu to ensure its wide availability. Although this system has led to high levels of stock, it is argued not to be desirable in the long as it leads to wastages when number of malaria patients is lower than expected (e.g. as a result of malaria control or the introduction of diagnostics). The National Malaria Control Programme (NMCP) has been progressively integrating the delivery of ALu into the national drug delivery system and by the beginning of 2010 all facilities should be placing orders for ALu along with other items (Dr Mkude, NMCP, personal communication). The impact on stock levels need to be closely monitored and evaluated.

Two other issues concerning the public health sector emerged from the retail audits: 1) the low rate of prescription of ALu; and 2) the non-adherence to user fee exemptions. Although ALu was widely available in public health facilities, it only accounted for 42% of anti-malarials dispensed in health facilities. Similar findings have been documented in Kenya [32,33]. In this study amodiaquine was still largely dispensed (32%), although the drug was no longer in stock (which does not exclude it from being in the dispensing room). These findings differ from information extracted from user surveys carried out a few months later (June to August) according to which 63% of patients who were treated in health facilities received ALu, 17% SP and 6% amodiaquine. A deliberate attempt by health facility staff to clear out any remaining stocks of amodiaquine in the first half of the year could be an explanation for this. This would be consistent with reasons documented in Kenya for low ALu prescription. Indeed, factors determining low prescription of ALu by health workers were not so much related to distrust in the drug, but more with concerns over the cost of ALu, fears of future stockouts and excess stocks of non-recommended anti-malarial drugs [32]. The data presented here clearly suggested a non-adherence to the user fee exemption given that half of the health facilities reported selling ALu packs for children under the age of eight. However, the finding is not conclusive as only four out of 10 health facilities provided pricing.

As far as the private sector is concerned both the availability and accessibility of drug shops substantially increased after the introduction of ADDOs. Interestingly, there is evidence that ADDOs were opened in less populated areas where there were previously no Part II drug shops, suggesting that the programme may have succeeded in encouraging shop owners to open ADDOs in more remote areas.

General shops no longer play an important role in the provision of anti-malarial treatment in the study area. A study carried out in 2001 found that 27% of the general shops selling drugs had anti-malarials in stock [3]. This study found that the proportion of general shops selling anti-malarial drugs decreased constantly between 2004 and 2008. There are a few possible explanations for this. On one hand this may be due to tighter supervision by district authorities as a result of the ADDO pilot in the area. However market mechanisms could also be operating. General shops may have been competed out of the market by the increase in the number of licensed drug shops or the increase in prices of anti-malarials.

The use of the private retail sector for malaria treatment increased despite increases in prices and much higher prices than in health facilities. Although prices of anti-malarials alone do not serve as an indication of affordability, price changes over a relatively short period of time provide a good indication of relative changes in affordability. The price of the first-line treatment for malaria increased by 84% and the price of SP by 51% in drug shops whereas both decreased by 20% in health facilities. Nevertheless, the share of doses sold in the private retail sector increased from 49% to 59%. There are many possible reasons behind this. Certainly improvements in availability and accessibility of shops must have contributed to their increased use. It might also be the result of greater demand for ADDO products compared to the previous Part II drug shops. Indeed, results from mystery shopper surveys conducted in parallel to the shop censuses suggest a major improvement in the quality of care in drug shops after the introduction of ADDOs. The percentage of mystery shoppers reporting with malaria symptoms increased from just above 30% to nearly 80% between 2004 and 2008 (Dillip et al., unpublished data). Another possible explanation could be the referral by health facility staff to shops in which they have a financial stake in, given that many ADDOs and Part II drug shops are owned directly or indirectly by health facility workers, but data from treatment-seeking surveys suggest that this was not a major problem in the study area [20].

Despite the many improvements seen, the low availability of ALu in ADDOs is a major concern. Most ADDO shop keepers reported selling ALu at its recommended retail price of TSH 500 (approximately USD 0.40) for children doses and TSH 1500 (approximately USD 1.30) for other doses. The results presented here indicate that this level of subsidy did not result in widespread availability of the drug. Discussions with ADDO dispensers highlighted two main reasons for this: 1) the drug can only be procured from one wholesaler in Morogoro, a city approximately 200 km from Ifakara; 2) the drug is too expensive and leads to low profit margins. A typical pre-packaged adult dose of SP was bought for TSH 900 (USD 0.80) and sold for TSH1500 (USD 1.30). Un-packaged SP was bought at the lower price of TSH 180 (USD 0.15) and sold for TSH 300 (USD 0.25). Conversely, a dose of ALu was bought for TSH 1150 (USD 1), which leaves a much lower profit margin than for other drugs. The experience from two studies which piloted the AMFm in Tanzania and Uganda where retail mark-ups were higher found higher stocking rates [34,35].

The findings presented here are highly relevant to the imminent roll-out of the AMFm and confirm that ensuring wide availability of ALu in the private retail sector is paramount to its uptake. ADDOs are a suitable distribution channel for ALu, since in the districts where they exist, they are available, accessible and highly utilized in both the district town and rural areas. The data presented here suggest that the low affordability of ALu in ADDOs ultimately restricted its availability. Cheaper drugs, such as amodiaquine and SP, were still the most dispensed drugs. AMFm plans to subsidize ACT high in the distribution chain and to facilitate their distribution at competitive prices along the same channels as other anti-malarials could present a viable solution to the problems encountered in the study area.

## Conclusions

The public and private retail sector are important complementary sources of malaria treatment in rural Tanzania and efforts to improve access to prompt and effective malaria treatment should take both sectors into account. This duality of sources also offers a backup solution when drugs are out of stock in the public heath system. A high proportion of patients seek treatment for malaria from shops rather than health facilities despite higher prices, most likely because shops are more available and more accessible. The introduction of subsidized ACT in the private retail sector was piloted in the study area through the ADDO programme but did not lead to high availability of the drug, probably because the final price was not affordable. This resulted in a low uptake of ACT despite its wide availability in public health facilities. If a high quality of services is ensured in the private sector the public health impact of ACT can be maximized by making this class of drugs available at affordable prices in the private sector.

## Competing interests

AS is employed by the Novartis Foundation for Sustainable Development (NFSD) which funded the ACCESS programme. The Foundation works independently from the company's business and supports not-for-profit health programmes in developing countries.

## Authors' contributions

SA coordinated the surveys between 2007 and 2008, analysed the data, drafted and finalized the manuscript. MWH coordinated the surveys between 2004 and 2006 and contributed to the manuscript. CG contributed to the coordination of the 2005 retail audit survey and contributed to the manuscript. AD contributed to the data collection and to the manuscript. JL coordinated the implementation of the ADDO programme and contributed to the manuscript. HM provided overall coordination and contributed to the discussion on the manuscript. CL contributed to the study design, the data analysis and the manuscript. The final manuscript was approved by all authors.
